# Enhanced functional connectivity and increased gray matter volume of insula related to action video game playing

**DOI:** 10.1038/srep09763

**Published:** 2015-04-16

**Authors:** Diankun Gong, Hui He, Dongbo Liu, Weiyi Ma, Li Dong, Cheng Luo, Dezhong Yao

**Affiliations:** 1 Key Laboratory For NeuroInformation of Ministry of Education, School of Life Science and Technology, University of Electronic Science and Technology of China, Chengdu 610054, China; 2 ARC Centre of Excellence in Cognition and its Disorders, Macquarie University, NSW 2109 Sydney, Australia; 3 School of Linguistics and Literature, University of Electronic Science and Technology of China, Chengdu 610054, China

## Abstract

Research has shown that distinct insular subregions are associated with particular neural networks (e.g., attentional and sensorimotor networks). Based on the evidence that playing action video games (AVGs) facilitates attentional and sensorimotor functions, this study examined the relation between AVG experience and the plasticity of insular subregions and the functional networks therein that are related to attentional and sensorimotor functions. By comparing AVG experts and amateurs, we found that AVG experts had enhanced functional connectivity and grey matter volume in insular subregions. Furthermore, AVG experts exhibited increased functional connectivity between the attentional and sensorimotor networks, and the experience-related enhancement was predominantly evident in the left insula, an understudied brain area. Thus, AVG playing may enhance functional integration of insular subregions and the pertinent networks therein.

Action video games (AVGs) are becoming increasingly popular worldwide. Similar to conventional sports (e.g., basketball, tennis), AVG playing requires a high level of attention and hand-eye coordination (read Supplemental materials for an example of an AVG). Given its influence on the development of cognitive functions, AVG experience has attracted increasing research attention recently^1^.

Behavioural research has shown that experienced AVG players have better attentional and sensorimotor functions than amateurs. For example, compared to amateurs, AVG experts exhibited improved selective attention on tasks of flanker compatibility, enumeration, useful field of view, and attentional blink; furthermore, AVG training improved participants' performance on the above tasks, thereby demonstrating the attentional effects of AVG playing^2^. Furthermore, AVG playing enhanced the spatial distribution of attention and attentional capture^3^,^4^, cognitive control^5^, and emotional regulation^6^. In addition, research on sensorimotor functions indicated that compared to amateurs, AVG experts had improved spatial resolution of vision^7^, multisensory temporal processing abilities^8^, hand-eye motor coordination^9^, contrast sensitivity^10^, oculomotor performance^11^, and body movement^12^.

Neuroscience research has also examined the neural basis of cognitive benefits of AVG experience. For example, AVG playing can change cortical networks for complex visuomotor transformation^13^, reduce the amplitude of visually evoked potentials during a non-voluntary attention condition^14^, and modulate P2 and P3 evoked potentials^15^. Furthermore, AVG experience is also associated with increased grey matter volume (GMV) in the dorsal striatum^16^,^17^, right posterior parietal cortex^18^, entorhinal cortex, hippocampus, occipital cortex^19^, right hippocampal formation, and right dorsolateral prefrontal cortex, as well as in both hemispheres of the cerebellum^20^.

However, little research has examined the relation between AVG experience and the plasticity of insula, an important brain area for attentional and sensorimotor functions. Insula contains multiple subregions and is related to various functions^21^. By examining resting-state functional connectivity (FC, which reflects the interaction among neuronal populations), Cauda et al. found that 1) the anterior insular subregions are involved in an anterior attentive network (A-network), which includes the superior, middle and inferior frontal gyri, the temporoparietal junction, the rostral anterior cingulate cortex, the cuneus, the precuneus, and the superior temporal gyri; 2) the posterior subregions are involved in a posterior sensorimotor network (P-network), which includes the sensorimotor, supplementary motor, superior temporal, middle temporal, lingual, and cerebellar cortices; and 3) a transitional subregion between the anterior and the posterior subregions^22^. Furthermore, the proposition that the anterior and posterior subregions are related to the A- and P-networks respectively was supported by probabilistic tract-tracing research^23^, meta-analyses^24^, and primate data^9^,^25^.

This study examined AVG-related effects on the plasticity of insular subregions and functional networks therein. We hypothesized that AVG experience is associated with an enhancement of insular subregions and A- and P-networks. This hypothesis was based on two aforementioned findings: AVG playing enhances both attentional and sensorimotor functions; and insular subregions and their functional networks play a crucial role in attentional and sensorimotor functions. Additionally, studies on neuroplasticity indicated that the FC of brain develops with age^26^ and can be adapted by learning activities^27^; furthermore, gaining experience can induce an increase of GMV in adults^28^ and the elderly^29^. We therefore used FC and GMV as measurements of insular function and structure, respectively.

To evaluate our hypothesis, we tested two groups of subjects: AVG experts and amateurs. The AVG experts were highly experienced AVG players who had at least 6 years of AVG experience and were recognised as regional or national champions. The amateurs did not play AVG habitually and had less than one year of AVG experience. We examined 1) the FC and GMV of ten bilateral subregions, 2) the FC of the A-and P-networks, and 3) the correlations among FC, GMV, and average weekly amount of time participants spent playing AVG (AT).

## Enhanced FC of insular subregions

**Figure 1:**
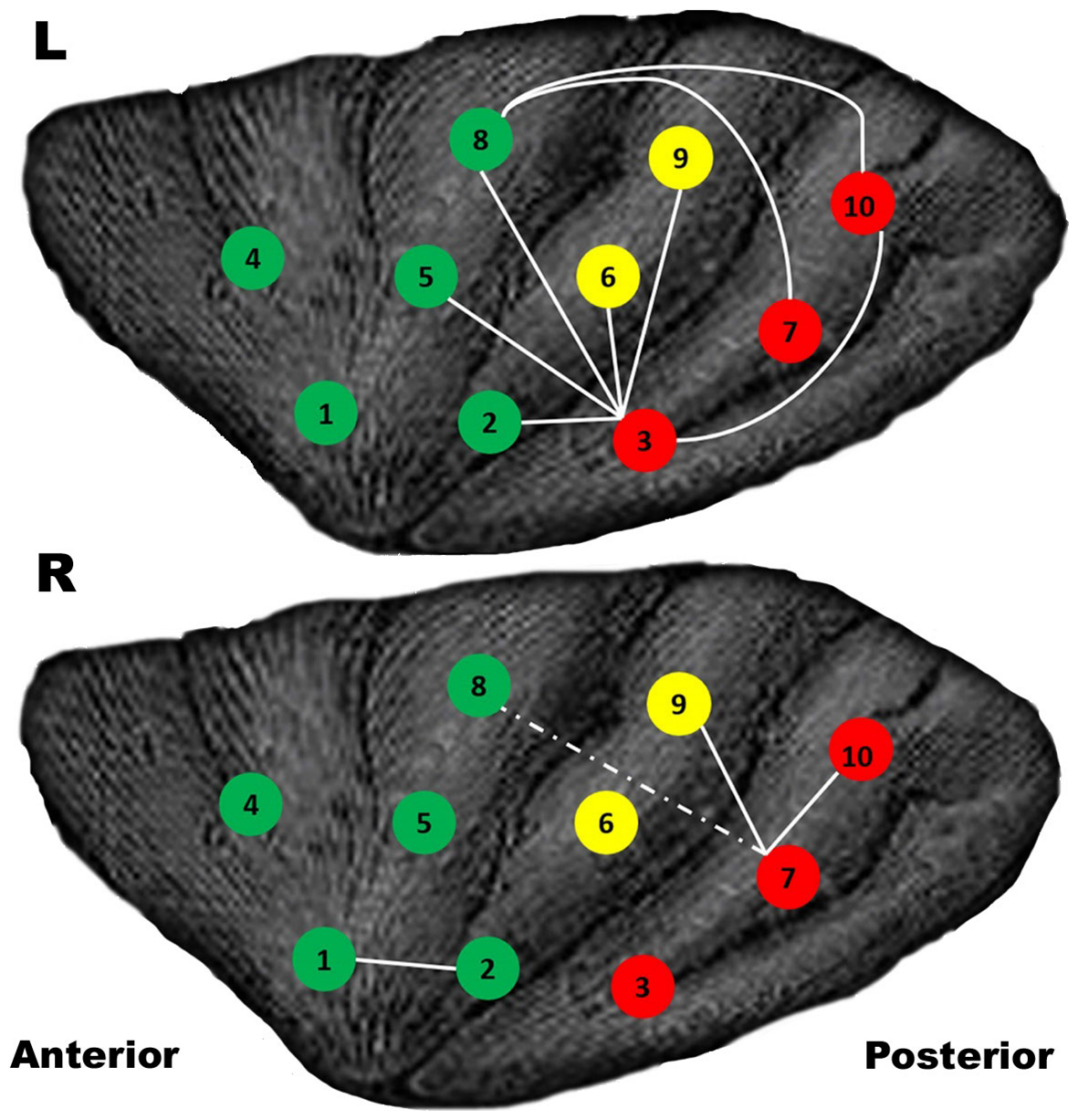
Enhanced FC of the insular subregions. The white lines denote the pathways where experts had significant enhancements compared with amateurs, *p* <0.05, FDR-corrected. The dashed line represents *p* <0.01, uncorrected. The higher FC reveals a pattern of anterior-posterior integration and left-lateralisation. ROIs 1, 2, 4, 5 and 8 (green) were located in the anterior subregions. ROIs 3, 7 and 10 (red) were located in the posterior subregions. ROIs 6 and 9 (yellow) were located in the transitional subregions.

**Figure 2:**
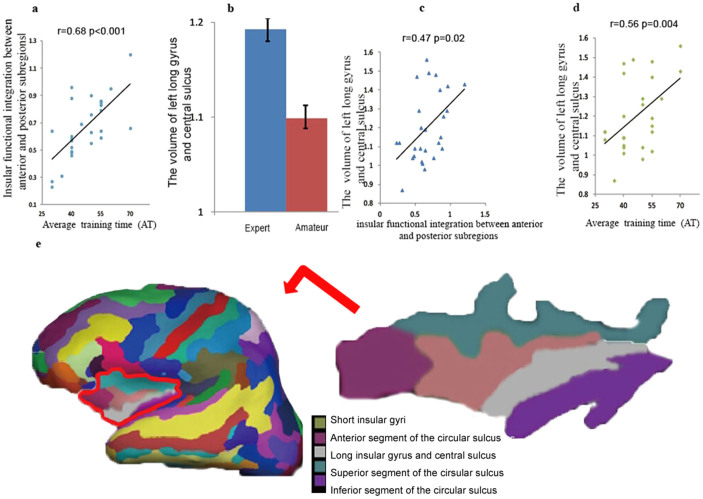
Correlation and GMV subregion analysis. 2a, 2b and 2c are pairwise Pearson correlations that include insular functional integration, GMV and the average playing time. 2d is a comparison between the two groups within the left long insular gyrus and central sulcus. 2e illustrates an inflated surface of the left hemisphere. The long insular gyrus and central sulcus is shown in grey and is located near the transitional subregion.

Compared with amateurs, AVG experts showed significantly higher FC between insular subregions. Furthermore, AVG experts with higher FC also showed anterior-posterior integration and left-lateralisation (Figure 1). The pattern of results is confirmed by insular functional integration analysis (described in Data Analysis), which showed greater insular functional integration in AVG experts than in amateurs [left: t(55) = 2.93, right: t(55) = 2.73, *p*'s <0.009]. Correlational analyses showed that only the left insular functional integration in experts was correlated with their AT (Figure 2a).

## Increased GMV of insular subregions

In line with the FC subregion analysis, a significant increase in GMV was observed in the left insula in experts (Figures 2b). Further analyses revealed an increased GMV in the short insular gyri, long insular gyrus, and central sulcus (Figures 2c). Correlational analyses showed that the increase of GMV in long insular gyrus and central sulcus of the left hemisphere were correlated with insular functional integration and AT in experts (Figures 2d). Correlational analyses in the amateur group did not reveal significant results.

## Enhanced FC of networks

**Figure 3:**
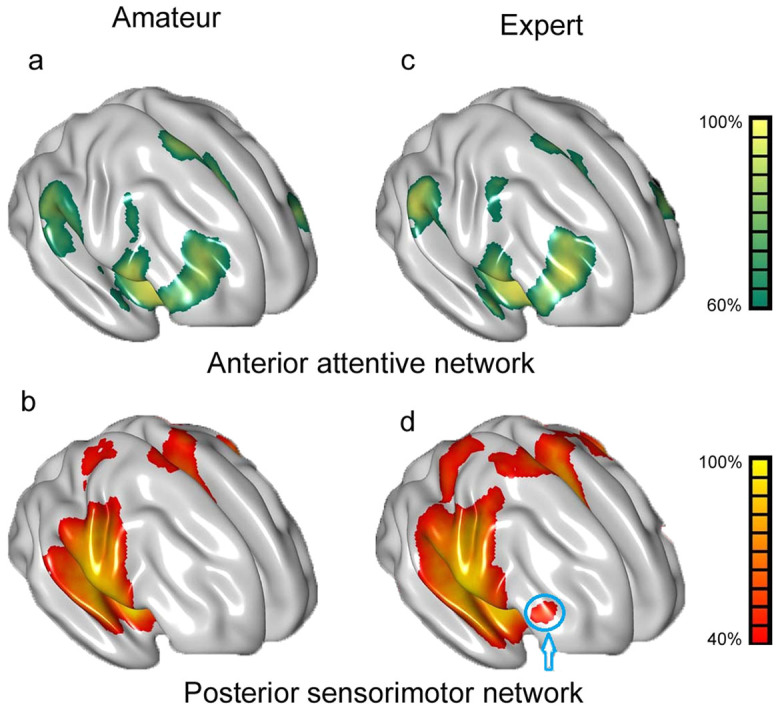
Examples of the A- and P-networks. The left ROI 4 is shown as a representative example in 3a and 3c, and the left ROI 10 is shown as a representative example in 3b and 3d. A spatial distribution similar to the left ROI 4 was observed in the other anterior ROIs, and a spatial distribution similar to the left ROI 10 was observed in the other posterior ROIs. See Supplemental Figure 1 for the analysis of other ROIs. Colours ranging from green to yellow or red to yellow indicate increasing spatial consistency (%). E.g., a 40% value in the amateur group indicates that the relevant brain region was activated in 12 subjects [40% = (12/30) × 100%]. The blue circle indicates the MFG, which is believed to be a key node in the A-network and was also identified in the P-networks of experts. The maps are projected onto a 3D brain surface using the BrainNet Viewer (http://www.nitrc.org/projects/bnv/).

In most ROIs, the amateurs' FC networks in this study were similar to their counterparts' in Cauda et el. (2010). ROIs 1, 2, 4, 5 and 8 showed a bilateral pattern (A-network) involving the superior, middle and inferior frontal gyri, as well as the bilateral temporoparietal junction, anterior cingulate, superior temporal gyri, and putamen (Figure 3a); ROIs 3, 7 and 10 also showed a bilateral pattern (P-network) linking the precentral gyri, postcentral gyri, superior temporal gyri, middle temporal gyri, supplement motor area, etc. (Figure 3b); and ROIs 6 and 9 exhibited the transition between the A- and P-networks (FC networks of all ROIs are presented in Supplemental Figure 1).

**Figure 4:**
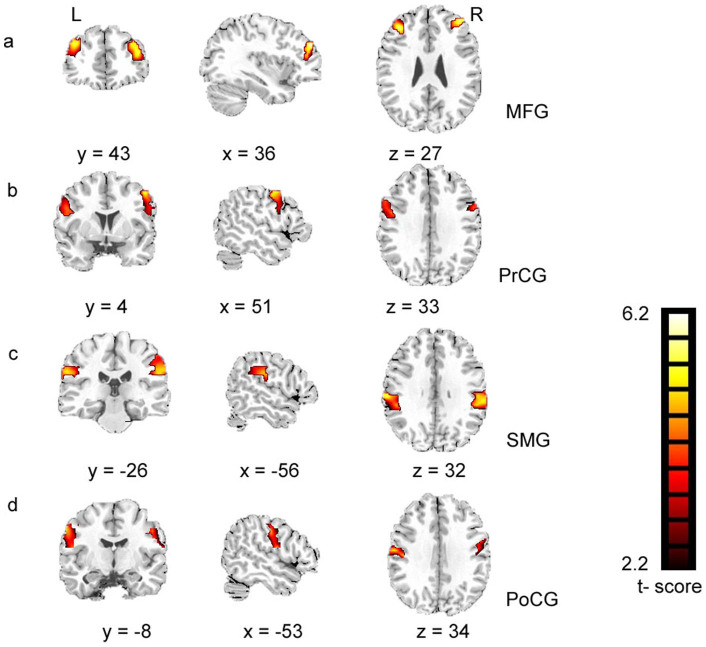
Examples of enhanced FC between groups (*p* <0.05, FDR-corrected, cluster threshold k> 20). These examples are based on a comparison of the results of the left ROI 3. Similar results were found in other posterior ROIs. SMG = supramarginal gyrus, PrCG = precentral gyrus, PoCG = postcentral gyrus.

Furthermore, we found a similar pattern of results in experts and amateurs in the anterior ROIs (Figure 3c) but not in the posterior ROIs (Figure 3d). In AVG experts, the ROIs within the posterior subregions were associated with the bilateral middle frontal gyrus (MFG), which was confirmed by the analysis of spatial consistency based on probability networks. The MFG is widely accepted as a key node in the A-network (blue arrow in Figure 3d). In addition, FC was enhanced within the A- and P-networks in the left vs. right insula of experts (Figure 4). See Supplemental Tables 1 and 2 for details.

## Discussion

Previous studies have shown that AVG playing is associated with improved performance on tasks that demand attention and/or sensorimotor abilities (e.g., AVG experts exhibit better visual selective attention, visuospatial attention, multisensory temporal processing abilities and hand-eye motor coordination). Furthermore, the anterior and posterior subregions of the insula are involved in an attentional network (A-network) and a sensorimotor network (P-network), respectively.

### Enhanced functional integration of subregions

This study found enhanced insular functional integration between the anterior and posterior subregions of the experts, predominately in the left insula (refer to Figure 1). The finding that left insular functional integration was correlated with AT in experts suggests an AVG-related enhancement^21^. Since the anterior and posterior subregions are involved in attentional and sensorimotor functions respectively^22^, the enhancement observed in experts is consistent with the previous finding that attention is essential for sensorimotor functions^30^. This enhancement may serve as the neural basis of the improved coordination between attentional and sensorimotor functions in experts.

### Increased GMV in insular subregions

Longitudinal studies have revealed that acquiring knowledge^31^ and learning skills^28^ can enhance the activity of relevant brain areas. Accordingly, researchers proposed that the increased GMV in insula is induced by relevant learning activities. For example, Giuliani et al. found that the use of expressive suppression can predict the GMV of the anterior subregions^32^, and that the neural activity of the left anterior subregions can be modulated by voluntary regulation in a real-time manner^33^; patients with better stroke recovery exhibit greater activation of the insula^34^; insular enhancement is related to learning languages^35^ and music^36^; and the GMV of the left posterior insular subregions is related to compensatory sensorimotor function in the deaf, who rely more on visual-motoric representations than the normal hearing^37^. Converging neuroscience evidence therefore suggests that the insula, particularly the left side, is sensitive to certain learning activities. Given the importance of attentional and sensorimotor functions in learning^21^, it is highly likely that the insula is enhanced by long-term AVG playing. The present study supports the above speculation by revealing increased GMV in the left long insular gyrus and central insular sulcus (Figure 2b). Further evidence demonstrated that only the GMV of the left long insular gyrus and central insular sulcus was correlated with insular functional integration and AT in the experts (Figures 2a and 2b).

Why is the long insular gyrus and central insular sulcus related to insular functional integration? This region is located near the transitional subregion (Figures 1 and 2e)^19^ between the anterior and the posterior subregions. Thus, the increased GMV of the long insular gyrus and central insular sulcus might enhance the functional integration between the anterior and posterior subregions. However, this is merely a conjecture yet to be tested by experimental studies.

### Enhanced functional integration in the insular networks

Can the enhancement in the insular subregions influence the functional networks therein? This study suggests that it can, based on the results of two comparisons: amateurs in this study vs. Cauda et al.'s study and anterior and posterior subregions in amateurs vs. experts.

First, we found a pattern of results similar to Cauda et al., such that the anterior and posterior subregions of amateurs contained distinct networks (Figures 3a and 3b). This finding supports Cauda et al.'s proposition that the anterior and posterior subregions are involved in the A- and P-networks respectively, which is also in accordance with previous findings on WM^23^, active task^24^, and anatomy^38^.

Second, we found a similar pattern of results in experts and amateurs in anterior subregions (Figure 3c), such that the enhanced FC had no effects on the A-network. However, we found significant differences between experts' and amateurs' posterior ROIs (Figure 3d). The bilateral MFGs were linked to posterior ROIs in experts, while they tended to be linked to anterior ROIs in amateurs. The finding was confirmed by a spatial consistency analysis (Figure 3d). The results suggested that enhanced FC in the subregions might influence the P-network pattern in experts. Given the results of GMV and FC of the subregions, we propose that the activity observed in the MFG in experts may indicate an enhanced functional integration between A- and P-networks. Furthermore, the subregional adaptation may serve as the neural basis of the functional integration between A- and P-networks.

**Figure 5:**
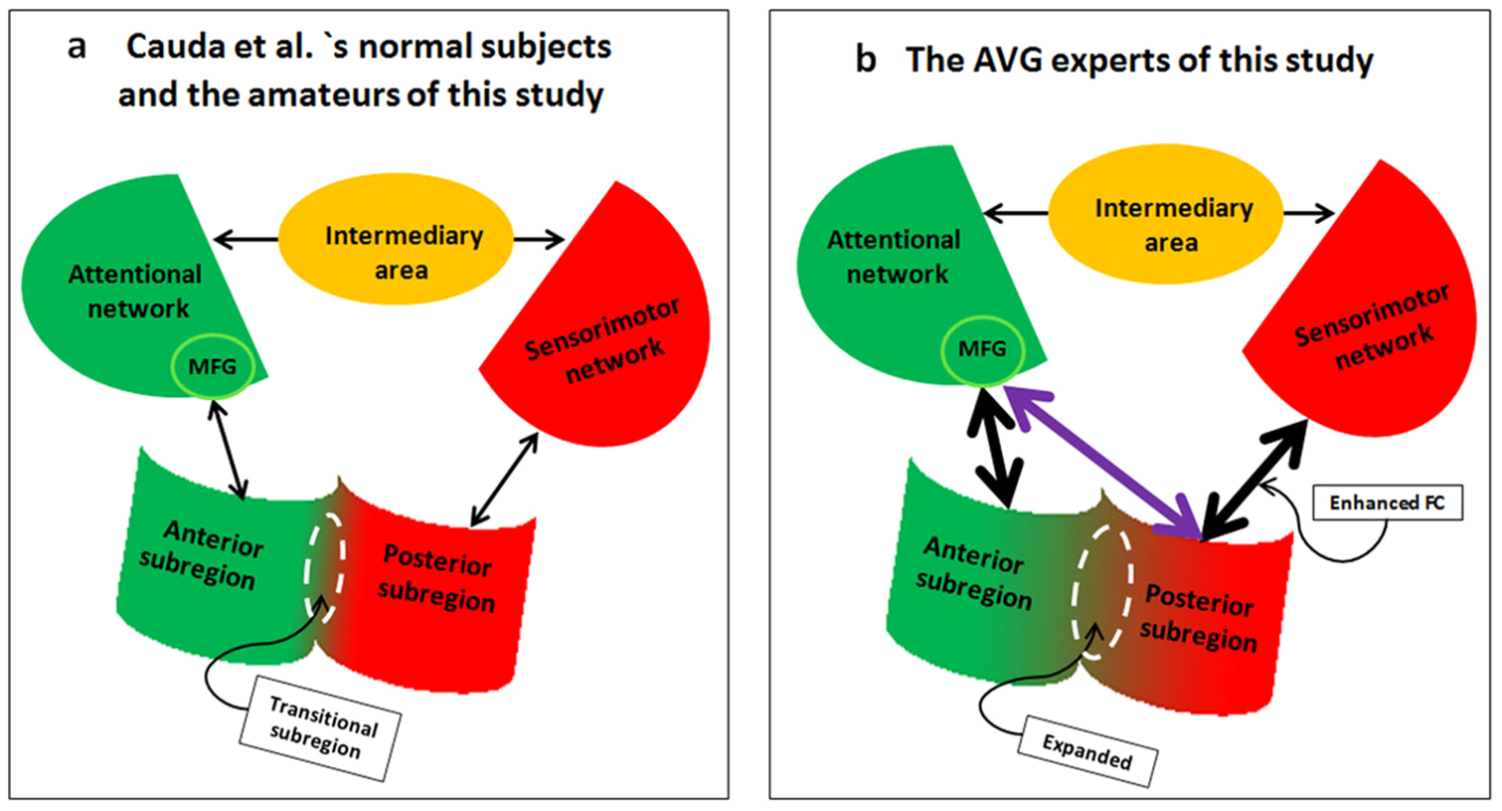
A summary of the findings of this study. The shapes represent brain areas (MFG, insula and a putative intermediate brain area). The lines with two arrowheads represent FC. 5a corresponds to the pattern of the insular network of the amateurs in this study and in Cauda et al.'s study 5b corresponds to the pattern observed in the insular network of the experts. Note that 5b shows an expanded transitional subregion (the dotted line circle) and a direct connection between the MFG and posterior subregion (purple line).

In addition, although the MFG is usually classified as a node of the A-network^22^,^23^,^39^,^40^, evidence has shown that the MFG is involved in control networks and is activated in higher-level functions, such as attention, spatial and episodic memory, and explicit contingency awareness^41^,^42^,^43^,^44^. The MFG should also be involved in the sensorimotor network even in amateurs^45^. However, studies have not found significant FC between the MFG and the sensorimotor networks in amateurs, which is perhaps due to the fact that the path length between the MFG and the sensorimotor networks is greater in amateurs than in experts^46^. Thus, the intermediate brain area, which connects the MFG and the sensorimotor networks, may decrease the possibility of detecting significant FC. Here, path length refers to the distance from one node to another in a network. A shorter path reflects fewer intermediate areas and indicates an increased interactive efficiency between nodes^47^. See Figure 5 for a summary of the findings of this study.

### Why does a AVG expert's insula surpass an amateur's

Attention is a determinant mechanism in sensorimotor functions^48^. In a typical AVG game, players may complete approximately 150 sensorimotor responses per minute using the keyboard and mouse, which requires coordination between attention and sensorimotor functions. As aforementioned, the anterior and posterior insular subregions are involved in attentive and sensorimotor networks respectively. AVG playing therefore demands for integration between attentive and sensorimotor networks, which may induce neural plasticity in the insula.

### Conclusion

By comparing AVG experts and amateurs, this study found enhancements in the AVG experts' FC between anterior and posterior insular subregions, GMV in the long insular gyrus and central insular sulcus, and functional integration between the attentional and sensorimotor networks. Furthermore, experience-based enhancement was predominately evident in the left insula. These results suggest that AVG playing may induce functional integration of insular subregions and pertinent networks therein. Currently, a longitudinal experimental study is examining the causal relation between AVG playing and neuroplasticity.

## Methods

### Subjects

The study protocol was approved by the ethics research committee at the University of Electronic Science and Technology of China (UESTC) and has been performed in accordance with ethical standards outlined by the Declaration of Helsinki. Informed consent was obtained from all subjects. A total of 27 AVG experts (*mean age* = 23.26 ± 0.4 years) and 30 amateurs (22.3 ± 0.38 years) participated in this study. All of the subjects were right-handed according to the Edinburgh Inventory^49^, reported normal or corrected-to-normal vision, had normal hearing and reported no history of neurological illnesses.

The experts were highly experienced players of AVGs (i.e., League of Legends [LOL] or Defence of the Ancient 2 [DOTA 2]). They had received AVG training for at least six years and were recognized as either regional or national champions in AVG competitions. The amateurs did not play AVG habitually and had less than one-year AVG experience. The experts' AVG experience was quantified based on their professional score, ranging from 1900 to 2600 points, measured on Elo's chess-skill rating scale^50^. The amateurs had less than 1200 points. The Elo rating scale is widely used as a rating system for multiplayer competition in AVGs. The difference in the ratings between two players serves as a predictor of the outcome of a match. A difference of 100 points indicates that the probability of winning a AVG match for the stronger player is 64%, 200 points is 76%. In general, an AVG expert has a rating of 1800 points or higher, while an amateur has a rating of approximately 1200 points.

Confounding variables were matched between groups (see Table 1, also see Du et al., 2011)^51^. The only significant between-group differences were average weekly amount of time spent on AVG Playing (AT) and game type (*t* = 12.39, *χ*^2^ = 20.2 *p*'s <0.0001), which further verified the group membership (i.e., experts *vs.* amateurs). Among the various behavioral measurements we used to gauge the experts' AVG experience, the experts' AT, appeared to be the most sensitive indicator to their AVG capability, since the experts' professional scores and performance on Dodge The Squares were correlated with their AT (*r*_scores_ = 0.41, *r*_performance_ = 0.45, *p*s <0.03).

### Data acquisition

Images were acquired on a 3T MRI scanner (GE Discovery MR750) at the MRI research centre of UESTC. Resting state functional MRI data were acquired using gradient-echo EPI sequences (repetition time [TR] = 2000 msec, echo time [TE] = 30 msec, flap angle [FA] = 90°, matrix = 64 × 64, 3 × 3 × 3 mm voxels, field of view [FOV] = 24 × 24 cm^2^, slice thickness/gap = 4 mm/0.4 mm), with an eight channel-phased array head coil. All of the subjects underwent a 510 sec resting state scan that yielded 255 volumes (32 slices per volume). High-resolution T1-weighted images were acquired using a 3-dimensional fast spoiled gradient echo (T1-3D FSPGR) sequence (TR = 6.008 msec, TE = 1.984 msec, FA = 9°, matrix = 256 × 256, FOV = 25.6 × 20 cm^2^ [80%], slice thickness [no gap] = 1 mm) to generate 152 slices.

### Data analysis

Data analysis was divided into two sections, FC analysis and GMV analysis. The FC analysis implicated both subregions and functional networks, and used an identical *data pre-processing* procedure. The FC subregion analysis was computed on pair-wise subregions, whereas the FC functional network analysis was conducted between subregions and voxels outside of the insula. The GMV analysis was performed only to examine subregions of interest.

### Functional MRI data pre-processing

Functional MRI data pre-processing followed typical pre-processing procedures using SPM8 (Wellcome Department of Cognitive Neurology, London, UK) and customised Matlab scripts. These procedures included discarding the first five volumes of each run, slice scan time correction, head motion correction^52^, and image normalisation using an EPI template from the Montreal Neurological Institute (MNI) atlas space. Spatial smoothing was applied with a Gaussian kernel of 8 mm full-width half-maximum (FWHM). Temporal filtering (band-pass) was between 0.01–0.08 Hz, and the mean signal was removed.

### Defining regions of interest (ROIs)

To avoid preconceptions during the segmentation of subregions, we selected the coordinates used by Cauda et al. (2011). Following this publication, we defined our ROIs in an equispaced pattern. In short, twenty ROIs (left insula: 1–10, right insula: 1–10) were chosen in three horizontal planes that were left-right identical (Figure 1). Each ROI is 7 voxels, nearly spherical, and occupies 189 mm^3^.

### Pre-processing of FC analysis

FC was computed based on multiple regression analyses. Signals were extracted by averaging the time courses from each ROI. To reduce the effects of physiological signals^53^,^54^, nine covariates and six motion parameters were added to the regression analysis^55^,^56^.

### FC analysis of subregions and insular functional integration

Subregion FC analysis included two steps. The FC was first computed between the ROIs. The FC was then calculated between the average signal of the anterior ROIs and the average signal of the posterior ROIs. This latter step of FC analysis was used to investigate the functional integration between the anterior and posterior subregions (hereafter, this FC is simply referred to as insular functional integration).

### FC analysis of functional networks

For each ROI, an individual FC network was computed on a voxel-wise basis and corrected according to the false discovery rate (FDR) with p <0.05 and k> 20 voxels. To test the spatial consistency of the FC networks, the probability network was calculated in two steps. The significant voxels were first summed up, and the sum was then divided by the number of subjects. This analysis provides a type of population-based spatial consistency. For example, a 40% value in the amateur group indicated that the relevant brain region was activated in 12 subjects [40% = (12/30) × 100%]. Therefore, higher spatial consistency is thought to reflect increased robustness.

### GMV analysis of subregions

We used FreeSurfer software to conduct a GMV analysis of the subregions (version 5.0.0; http://surfer.nmr.mgh.harvard.edu). Previous work has validated FreeSurfer by comparing it to histological analysis^57^ and manual measurements^58^. The computational steps have been described in detail elsewhere^59^.

The automated procedure involves segmentation of the WM^60^, tessellation of the grey matter/white matter (GM/WM) junction, inflation of the folded surface tessellation patterns^61^, and automatic correction of any topological defects in the resulting manifold. This surface is then utilised as the starting point for a deformable surface algorithm designed to find the GM/WM and pial (GM/cerebrospinal fluid) surfaces with sub-millimetre precision (Fischl and Dale, 2000). For each subject, the cortical surface area and cortical thickness of the cortical ribbon were computed on a uniform grid (comprised of vertices). The cortical thickness is defined as the shortest distance between the GM/WM and pial surface models. The thickness maps produced are not limited to the voxel resolution of the image and are therefore sensitive to sub-millimetre differences between any two groups being compared^62^. Thickness, surface area, and volume measures were mapped onto the inflated surface of each subject's brain reconstruction, allowing visualisation of the data across the entire cortical surface (i.e., gyri and sulci) without being obscured by cortical folding. The reconstructed brain image for each subject was then morphed into an average spherical surface representation that optimally aligned the sulcal and gyral features across subjects^61^. This procedure accurately matches the morphologically homologous cortical locations among subjects based on their individual anatomy. Finally, maps of the thickness, surface area, and volume were created. One of the implemented parcellation schemes in FreeSurfer (aparc.a2009s) was used to compute the five subregions of the insula (short insular gyri, anterior segment of the circular sulcus, long insular gyrus and central sulcus, superior segment of the circular sulcus and inferior segment of the circular sulcus). The entire cortex of each participant was visually inspected, and segmentation inaccuracies were manually corrected.

### Correlation analysis and comparison between groups

To investigate whether the brain enhancement observed in experts is correlated with AVG playing, we computed pairwise Pearson correlations that included insular functional integration, GMV and the AT. An independent sample *t*-test was performed between groups. Multiple comparisons were corrected according to the FDR with *p* <0.05 and a cluster threshold for the FC maps of k> 20.

## Supplementary Material

Supplementary information Supplementary information

## Figures and Tables

**Table 1:** The subject variables and behavioral performance (Mean ± Std.E)*

Group	RPM	Ef	Onset	Current AT	Gt	Ds
	(%)	(per month)	age	(per week)	AVG/RPG/Both	(sec.)
Expert	90±10.28	2.4±.21	8.03	46.67±2.1	27//	25.2±3.2
Amateur	90.2±9.8	2.1±.14	8.9	14.2±1.1	14/4/12	15.4±3.5

*RPM = Raven's Progressive Matrices, Ef = physical exercise frequency per month, Onset age = the onset age of playing video games, AT = the average weekly amount of time (in hours) spent on AVG playing, Gt = game type, AVG = Defence Of the Ancients or League Of Legends (two specific AVGs played by the experts), RPG = role play games (unlike the AVG, the RPG does not require high reaction speed and good hand-eye coordination under stringent time pressure. In a RPG, a player assumes the roles of characters in a fictional setting and takes responsibility for acting out these roles through a process of structured decision-making or character development.), Ds = Dodge The Squares (Players move a red block within a box to avoid crashing into blue blocks and touching the edge of the box. This game was used to further verify the group membership between experts and amateurs. http://www.gamemew.com/skill-games/square-dodge.html).
