# Supplementary material for: Enhanced functional connectivity and increased gray matter volume of insula related to action video game playing
Source: Sci Rep. 2015 Apr 16;5:9763. doi: 10.1038/srep09763 (PMC5381748; doi:10.1038/srep09763)
Supplement: Supplementary information [file srep09763-s1.pdf]

Enhanced functional connectivity and increased gray matter volume of insula related to action video game playing

Diankun Gong1, Hui He1*, Dongbo Liu1*, Weiyi Ma1, 2, 3*, Li Dong1, Cheng Luo1, Dezhong Yao1#

1Key Laboratory For NeuroInformation of Ministry of Education, School of Life Science and Technology, University of Electronic Science and Technology of China

2ARC Centre of Excellence in Cognition and its Disorders, Macquarie University, NSW 2109 Sydney, Australia

3School of Linguistics and Literature, University of Electronic Science and Technology of China, Chengdu 610054, China

An example of an AVG

In League of Legends, a player controls a single champion unit who wields multiple abilities. E.g., the champion named *Garen* has five unique abilities: perseverance, decisive strike, courage, judgment and justice. Typically, many practice attempts are required to learn where and when to use each ability. There are hundreds of champions available for deployment. Playing this AVG involves two teams, each consisting of five players (one forward, two centre forwards, and two shooting guards); the players fight on teams against teams of computer-controlled units with the goal of eliminating each other. To win, the players need to know multiple champions and battlefield landforms (involving memory and perception), constantly make tactical plans (involving attention and working memory). Additionally, the players must manipulate the champions using the keyboard and mouse with over 150 bimanual actions per minute (involving attention and sensorimotor integration). Defense of the Ancients is another popular AVG and has game mechanics that are nearly identical to those of LOL.

Supplemental figure

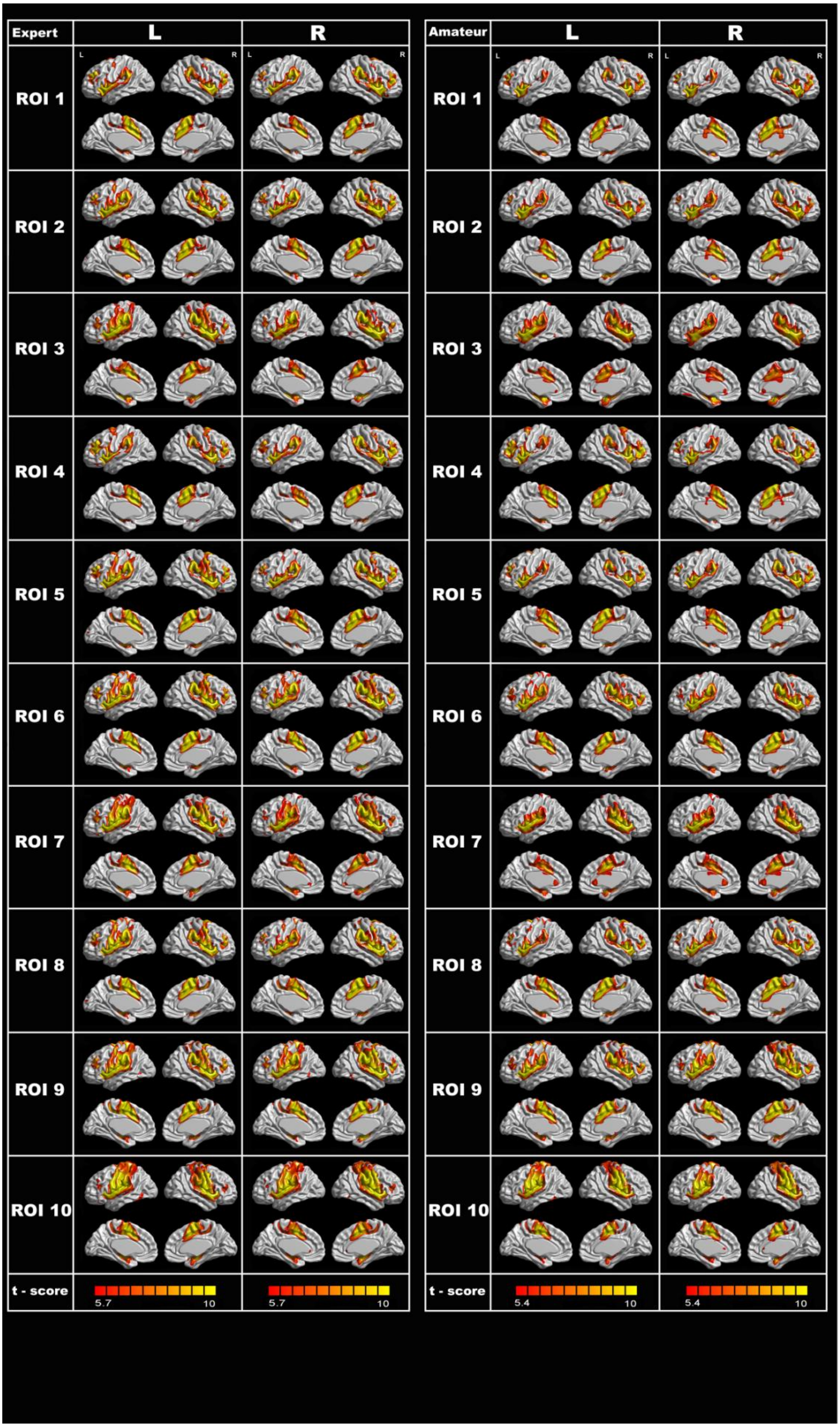
Expert
L
R
Amateur
ROI 1
ROI 2
ROI 3
ROI 4
ROI 5
ROI 6
ROI 7
ROI 8
ROI 9
ROI 10
t - score
5.7
10
5.4

Supplemental figure 1.

One sample t-test maps for all ROIs in two groups. For example, the upper left map is the map of left ROI 1. All maps are in p<0.05, FWE-corrected, cluster threshold k>20. Colors from red to yellow indicate an increasing t-value. Maps are projected on a 3D brain surface with the BrainNet Viewer (http://www.nitrc.org/projects/bnv/).

Supplemental tables

The insignificant results were not listed. For example, the first line of Table 2 listed the significantly enhanced FC between the left ROI 2 and the left MFG.

Supplemental table 1 The enhanced FC of left insular networks ($p < 0.05$, FDR-corrected, cluster threshold k > 20)

**Experts V.S. Amateurs**	**Anatomical location (AAL template)**	**Voxels**	**MNI coordinate [X Y Z]**	***t*-score**
ROI 2	Frontal_Mid_L	124	[-36 39 32]	4.391
	Frontal_Mid_R	89	[33 45 30]	3.787
	Supp_Motor_Area_L	87	[-3 6 54]	3.652
	Supp_Motor_Area_R	22	[3 6 54]	3.359
	SupraMarginal_L	104	[-57 -30 33]	3.016
	SupraMarginal_R	34	[53 -33 36]	3.222

	Parietal_Inf_L	65	[-51 -33 39]	3.153
	Rolandic_Oper_L	41	[-42 -6 15]	3.205
ROI 3	Frontal_Mid_L	125	[-39 39 33]	5.661
	Frontal_Mid_R	141	[36 45 30]	5.937
	Supp_Motor_Area_L	206	[-3 3 54]	5.307
	Supp_Motor_Area_R	245	[6 3 54]	5.799
	SupraMarginal_L	243	[-49 -39 30]	3.752
	SupraMarginal_R	334	[57 -33 30]	4.283
	Postcentral_L	507	[-51 -15 33]	4.087
	Postcentral_R	294	[51 -12 36]	3.201
	Precentral_L	214	[-54 3 30]	3.614
	Precentral_R	220	[48 -3 45]	5.15
	Parietal_Inf_L	209	[-51 -30 42]	3.378
	Rolandic_Oper_L	224	[-45 -6 15]	4.52
	Rolandic_Oper_R	205	[54 -6 15]	3.043
	Temporal_Sup_L	120	[-48 -36 21]	3.929
	Temporal_Sup_R	92	[57 -27 18]	3.516
	Temporal_Inf_R	30	[54 -57 -3]	3.732
	Insula_L	222	[-33 12 9]	4.421
	Insula_R	123	[39 9 6]	3.693

ROI 6	Frontal_Mid_L	87	[-39 38 33]	4.148
	Frontal_Mid_R	60	[36 45 30]	4.034
	Supp_Motor_Area_L	96	[-6 3 54]	4.165
	Supp_Motor_Area_R	30	[3 6 54]	4.395
	Postcentral_L	32	[-42 -14 45]	4.526
ROI 7	Frontal_Mid_L	116	[-38 39 33]	5.1
	Frontal_Mid_R	88	[42 43 18]	4.375
	Supp_Motor_Area_L	135	[-3 3 57]	4.575
	Supp_Motor_Area_R	174	[6 3 57]	4.625
	SupraMarginal_L	50	[-60 -27 27]	3.075
	SupraMarginal_R	106	[54 -36 30]	3.55
	Precentral_L	198	[-54 3 24]	3.25
	Precentral_R	63	[48 -3 48]	3.725
	Postcentral_L	107	[-42 -14 48]	4.075
	Parietal_Inf_L	73	[-51 -36 51]	3.35
	insula_L	147	[-33 15 9]	4.1
ROI 8	Frontal_Mid_L	50	[-39 36 31]	3.225
	SupraMarginal_L	121	[-60 -30 33]	4.044
	Insula_L	82	[-39 -6 0]	3.8
	Insula_R	24	[43 -2 1]	3.605

	Rolandic_Oper_R	24	[57 -9 12]	3.28
	Rolandic_Oper_L	59	[-42 -9 18]	4.17
ROI 9	Frontal_Mid_L	50	[-39 39 32]	3.457
	Frontal_Mid_R	55	[36 45 30]	3.722
	Supp_Motor_Area_L	93	[-6 3 54]	4.166
	Supp_Motor_Area_R	39	[3 6 54]	4.478
	Rolandic_Oper_L	36	[-42 -12 18]	4.166
	Insula_L	33	[-39 -6 -3]	4.601
	Insula_R	43	[45 0 -3]	3.845
	Postcentral_L	60	[-45 -15 42]	3.977
ROI 10	Frontal_Mid_L	32	[-36 41 22]	4.331
	Supp_Motor_Area_L	103	[-3 3 54]	4.088
	Supp_Motor_Area_R	115	[6 3 54]	3.694
	SupraMarginal_L	37	[-61 -27 30]	3.263
	SupraMarginal_R	51	[54 -36 30]	3.3
	Temporal_Sup_L	39	[-60 -39 15]	3.15
	Insula_L	117	[-33 18 6]	3.769

Supplemental table 2 The enhanced FC of right insular networks ($p < 0.05$, FDR-corrected, cluster threshold k > 20)

Expert V.S. Amateur	Anatomical location (AAL template)	Voxels	MNI coordinate [X Y Z]	*t*-score
ROI 2	Frontal_Mid_L	94	[-42 39 32]	4.048
	Frontal_Mid_R	101	[36 45 30]	2.979
	Supp_Motor_Area_L	97	[-3 3 53]	3.216
	Supp_Motor_Area_R	35	[6 9 54]	3.246
	SupraMarginal_L	131	[-57 -30 33]	3.632
	SupraMarginal_R	120	[54 -33 36]	3.276
	Rolandic_Oper_L	67	[-43 -6 15]	3.842
	Insula_L	58	[-39 -3 9]	3.172
ROI 3	Frontal_Mid_L	30	[-42 41 21]	2.994
	Frontal_Mid_R	38	[45 45 15]	3.851
	Supp_Motor_Area_L	106	[-3 -3 57]	3.234
	Supp_Motor_Area_R	157	[6 6 54]	3.372
	SupraMarginal_L	156	[-61 -26 32]	3.138
	SupraMarginal_R	226	[52 -31 42]	3.677
	Postcentral_L	178	[-60 -15 30]	3.965
	Postcentral_R	154	[54 -12 32]	3.007
	Precentral_L	64	[-54 6 33]	3.385
	Precentral_R	114	[57 6 33]	3.914

	Rolandic_Oper_L	158	[-45 -6 15]	4.053
	Rolandic_Oper_R	166	[48 0 15]	3.234
	Temporal_Inf_R	29	[54 -57 -3]	3.435
	Parietal_Inf_L	107	[-54 -30 48]	3.335
	Temporal_Sup_L	41	[-61 -9 3]	3.309
	Temporal_Sup_R	41	[60 -3 0]	3.095
	Insula_L	131	[-39 -3 9]	3.574
	Insula_R	50	[37 3 12]	3.057
ROI 6	Frontal_Mid_L	91	[-42 39 32]	4.258
	Frontal_Mid_R	97	[36 45 30]	3.012
	Supp_Motor_Area_L	135	[-3 3 54]	4.235
	Supp_Motor_Area_R	139	[9 6 54]	3.986
	SupraMarginal_L	150	[-57 -27 27]	3.782
	SupraMarginal_R	139	[63 -21 27]	3.082
	Precentral_R	146	[48 -3 45]	3.465
	Postcentral_L	184	[-54 -15 30]	3.238
	Insula_L	75	[-36 -3 12]	3.306
	Rolandic_Oper_L	75	[-42 -3 15]	2.944
	Rolandic_Oper_R	83	[48 -6 18]	2.808
ROI 7	Frontal_Mid_R	10	[41 43 15]	4.214

Supp_Motor_Area_L	108	[-3 3 54]	3.767
Supp_Motor_Area_R	171	[6 6 54]	3.833
SupraMarginal_L	36	[51 -36 30]	3.441
Postcentral_L	364	[-57 -12 27]	3.233
Postcentral_R	166	[57 -6 33]	3.061
Precentral_R	189	[51 -3 48]	3.269
Insula_L	98	[-33 15 9]	3.024
Insula_R	95	[36 21 6]	3.063
